# Targeting the parietal memory network with tDCS in MCI: study protocol for a randomized controlled trial

**DOI:** 10.3389/fnhum.2025.1661790

**Published:** 2025-11-10

**Authors:** Seyda Cankaya, Aynur Akturk, Ayse Karakus, Lütfü Hanoğlu, Adil Mardinoglu, Burak Yulug

**Affiliations:** 1 Department of Neurology and Neuroscience, Faculty of Medicine, Alanya Alaaddin Keykubat University, Alanya, Türkiye; 2 Department of Neurology and Neuroscience, Faculty of Medicine, Istanbul Medipol University, Fatih, Türkiye; 3 Science for Life Laboratory, KTH–Royal Institute of Technology, Stockholm, Sweden; 4 Centre for Host-Microbiome Interactions, Faculty of Dentistry, Oral and Craniofacial Sciences, King’s College London, London, United Kingdom

**Keywords:** tDCS, parietal memory network, mild cognitive impairment, Alzheimer’s disease, Parkinson’s disease

## Abstract

**Background:**

Mild cognitive impairment (MCI) is a critical transitional stage in dementia related disorders. In that context, dorsolateral prefrontal cortex (DLPFC), and lateral parietal cortex (LPC) are subjected to neuropathological changes in MCI. Furthermore, alterations in parietal memory network (PMN) integrity and default mode network (DMN) also occur in MCI. Transcranial direct current stimulation (tDCS) is a promising neuroprotective tool that might interfere with cognitive decline in Alzheimer’s disease-MCI (aMCI) and Parkinson’s disease-MCI (PD-MCI) when applied to DLPFC or LPC separately.

**Methods:**

This is a randomized and controlled study evaluating the effectiveness of tDCS in 120 patients (60 aMCI and 60 PD-MCI). Firstly, all patients will be randomly (1:1) divided into two groups: DLPFC (30 aMCI; 30 PD-MCI) and LPC (30 aMCI, 30 PD-MCI) for tDCS stimulation. Secondly, they will classify randomly (2:1) real and sham groups for tDCS applied to once a day for 10 days over 2 weeks. The stimulation will be delivered with a 2-mA current frequency and will last 20 min. The primary outcome assessment for this study will be the change in score from baseline to the end of (14-days and 90 days follow-up) the tDCS application for the neurocognitive tests. Potential outcome parameters will be discussed in the light of current literature to contribute to the new area of personalized non-invasive brain stimulation research in neurodegenerative diseases at early stages. The results of this study are expected to shed light on the neural underpinnings and pro-cognitive outcomes of tDCS. Potential outcome parameters will be discussed in the light of current literature to contribute to the new area of personalized non-invasive brain stimulation research in neurodegenerative diseases at early stages.

## Background

Mild cognitive impairment (MCI) is used as a term to describe the period between cognitively healthy aging and cognitively dysfunctional aging. Technically, MCI is defined as 1–1.5 standard deviations beneath the normative data in neuro-assessment for the same age group, which is suggested to be sufficient for accurate clinical diagnosis (Albert et al., 2011; Petersen, 2004; Petersen et al., 2014). There are two different sub-groups of MCI, depending on the cognitive domain affected. Amnestic MCI (aMCI) refers to the impairment in cognition when the memory area is predominantly affected, while non-memory domains if affected are categorized as non-amnestic MCI. However, irrespective of the heterogeneous clinical course described above, MCI is a critical transitional stage in which early interventions can be applied to slow the cognitive deterioration. Thus, the recent framework (Diagnostic and Statistical Manual of Mental Disorders: DSM-5) suggested that MCI, as a transition phase, both accompanies the Alzheimer’s disease (AD) processes, and other various degenerative neurological disease processes (American Psychiatric Association, 2022; Carver et al., 2014).

As in AD, MCI can also be observed in the initial stages of Parkinson’s disease (PD), presenting in 18.9–38.2% of PD cases (PD-MCI) characterized by critical deficits in cognitive subdomains (Litvan et al., 2012). Furthermore, there is convincing evidence that PD-MCI is predictive of PD-Dementia (Litvan et al., 2011).

Specific cognitive deficits occurring in the prodromal stage in AD-MCI and PD-MCI are also associated with several biomarker changes during the progression to dementia, particularly including the amyloid beta burden and Lewy Body pathology (Aarsland et al., 2021; Besser et al., 2016). Furthermore, despite some disease-specific alterations, there is convincing evidence showing that Lewy Body pathology and neurofibrillary degeneration are common pathologies reported in both PD-MCI and AD-MCI participants (Besser et al., 2016), making a clear-cut differential diagnosis difficult when considering only the biomarker changes in the early phases of both disorders. The same is also true for brain network changes; for instance, several common cognitive networks, such as the lateral parietal cortex (LPC), dorsolateral prefrontal cortex (DLPFC), default mode network (DMN), and parietal memory network (PMN), are affected during neurodegenerative disease progressions (Buckner et al., 2005; Celone et al., 2006; Gratwicke et al., 2015; Sestieri et al., 2011), including both PD and AD.

Among them, DMN, with its well-known role in several memory functions (Kwon et al., 2023; Sestieri et al., 2011) also belongs to a network known as the PMN (Gilmore et al., 2015; Kwon et al., 2023). PMN is a recently defined network, functionally dissociated from the DMN, not only in particular recall tasks (coding, retrieval of recognition memory) but also in resting states during the aging process, indicating its critical role in age-related dementia disorders (Hu et al., 2019). This aligns with recent literature suggesting that functional and integrity alterations involving DMN, PMN, and the posterior control network (PCN) play a critical role in cognitive performance in AD and PD-MCI (Gilmore et al., 2015; Hu et al., 2019; Sestieri et al., 2011).

These findings together support that PMN alterations, in addition to DMN and LPC, may represent a powerful imaging biomarker, increasing the predictive value for the detection of impairments in cognition at the initial stages of AD and PD. Considering all this evidence, stimulating relevant brain structures, particularly the PMN, with transcranial direct current (tDCS) may serve as an impactful modulation technique for inducing pro-cognitive effects against neural impairment, as suggested for DMN in MCI patients (Hanoglu et al., 2023; Hunter et al., 2015). This approach will be advantageous against the conventional framework for research purposes in non-symptomatic degenerative phases associated with cognitive deficits.

## Hypothesis

The main objective of this study is to strengthen the evidence for clinical efficacy of tDCS in patients with mild cognitive impairment, while simultaneously embedding a mechanistic neuroimaging and electrophysiological perspective, and comparing the clinical effectiveness to determine if repeated stimulation over the parietal memory network or the DLPFC will yield measurable differences in improvements in cognitive performances compared with sham stimulation. Our secondary hypotheses aim to evaluate whether these cognitive improvements will be mediated by modulation of network-level integrity in the PMN and DMN, as will be assessed through resting-state functional MRI (fMRI) and EEG connectivity measures.

Regarding the rTMS, there have been considerable recent studies that analyzed the efficacy and safety of rTMS and tDCS in individuals with cognitive impairment and patients with Alzheimer’s Disease and MCI, and even in healthy individuals. For instance, several rTMS studies (Hanoglu et al., 2022, 2023) are showing promising results in AD, MCI, and PD (Jung et al., 2024; Zhang et al., 2022). A recent meta-analysis of 22 studies (1,074 patients) provides a good example of the beneficial effect of rTMS in AD and MCI, confirming the results by Hou et al. (2024). Also, a very recent work by Jung et al. showed that hippocampus and precuneuss- targeted repetitive transcranial magnetic stimulation (rTMS) increased functional connectivity and improved cognitive test scores in early AD patients (Jung et al., 2024).

Among the various non-invasive neuromodulation tools, tDCS works by stimulating the neurons via modulation of membrane polarity through increasing and reducing their excitability via anodal and cathodal stimulation, respectively (Angelakis et al., 2014; Bourdillon et al., 2019; Yamada and Sumiyoshi, 2021). In pathophysiological terms, tDCS application alters the signaling and transmission of various neurometabolites, which correlates with specific regional brain activity. tDCS is also used for the enhancement of cognition and emotional regulation (Hunter et al., 2015; Yamada and Sumiyoshi, 2021) associated with reversible reorganization of the altered whole-brain connectivity on both functional and structural levels. In light of the above, it appears reasonable to hypothesize that anodal tDCS stimulation of DLPFC and LPC may result in improved memory skills and executive functions in PD-MCI and AD-MCI patients, which may be associated with critical DMN, PMN and hippocampal connectivity changes. Within that context, several studies have suggested the pro-cognitive effects of tDCS in PD-MCI and AD-MCI patients (Angelakis et al., 2014; Brak et al., 2022; Ferrucci et al., 2008; Liu et al., 2021; Marceglia et al., 2016), a fact which has already been suggested in patients at dementia stage. For instance, a recent meta-analysis highlighted that anodal tDCS and 20 Hz TMS significantly improved cognitive function in AD by modulating neural activity (Andrade et al., 2024). Also, a notable example is a recent study that showed a significant improvement in age-related cognitive decline (Flöel, 2014).

Additionally, other studies have confirmed tDCS’s role in enhancing cognitive task performance, reducing pathological brain activity, and improving disturbed brain connectivity patterns in patients at the dementia stage (Boggio et al., 2009; Meinzer et al., 2015). The beneficial role of tDCS in AD has also been confirmed by a very recent study by LoBue et al. that high definition tDCS produced meaningful cognitive enhancements in a proportion of patients having AD with improvements maintained for at least 8 weeks in some when targeting the medial prefrontal cortex (LoBue et al., 2025). It is also worth mentioning that the beneficial effects of tDCS on cognitive function have also been demonstrated in healthy individuals. For example, a study on healthy adults showed improved visuospatial working memory performance after anodal tDCS (Jeon and Han, 2012).

Given that tDCS exhibits similar pro-cognitive effects in patients with dementia (Bystad et al., 2016; Rasmussen et al., 2021), it may be advantageous due to its properties, such as being user-friendly, relatively inexpensive, and well-tolerated compared to similar non-invasive brain stimulation devices like rTMS (Mehta et al., 2024; Vanneste et al., 2010). Furthermore, no serious side effects have been reported with tDCS, supporting its use in cognitive research on early dementia. Although recent evidence indicates a greater efficacy in combined interventions involving neuromodulation and cognitive therapy, this combinatory approach has some caveats, such as the standardization of the cognitive therapy, the quality and quantity of well-educated cognitive therapists as well as tDCS stimulation side and sessions (Horczak et al., 2023; Lau et al., 2024; Lawrence et al., 2018).

There are limited studies of tDCS and cognitive therapy (CT) applied in the early stages of dementia (de Sousa et al., 2020; Gonzalez et al., 2021; Inagawa et al., 2019; Lu et al., 2019; Martin et al., 2019; Rodella et al., 2022) compared to relatively rich literature on rTMS and AD studies. For instance, a recent meta-analysis suggested that tDCS combined with CT offers significant advantages in improving language function in MCI (Yang et al., 2024). However, a recent novel study conducted by Antonenko et al. showed that cognitive therapy sessions with concurrent tDCS application did not lead to a cognitive improvement in individuals with cognitive impairment leading again to the questioning of the effect of cognitive training in this population (Antonenko et al., 2024). This has also been suggested by a recent study, which shows only significant connectivity changes in the combination group without a significant behavioral effect (Šimko et al., 2024). Finally, very recent a systematic review and meta-analyses covering 3,828 studies revealed that there was no difference of the impact of tDCS and CT versus tDCS alone indicating even better outcomes in the tDCS alone arm (Prathum et al., 2025).

To conclude, several studies have suggested the pro-cognitive effects of tDCS in PD-MCI and AD-MCI patients (Angelakis et al., 2014; Brak et al., 2022; Ferrucci et al., 2008; Liu et al., 2021; Marceglia et al., 2016). However, methodologically, there is no head-to-head study comparing the effects of tDCS in both disorders simultaneously. Furthermore, these studies are limited to specific areas of application, which miss the beneficial effects of stimulating the novel PMN network. Additionally, despite strong design and powerful analysis methods, these trials included only a limited number of participants.

In light of the above gaps, the primary aim of the current study is to explore the modulatory effects of tDCS application over the DLPFC and LPC regions. The secondary aim is to evaluate the network and cognitive correlates of tDCS, with a focus on the PMN, DMN network, and hippocampal connectivity properties.

## Evaluation of the hypothesis

As briefly mentioned above, the primary endpoint of this study is the effect of anodal tDCS on cognitive improvement in patients with MCI, as measured by changes in performance on a standardized neurocognitive test battery. Composite *z*-scores will be derived for parietal memory network–related memory measures (primary composite outcome) and executive-function tasks (key secondary cognitive outcomes). Cognitive assessments will be performed at baseline, immediately after the intervention (Day 14), and at follow-up (Day 90).

The secondary endpoints are the neural correlates of stimulation-induced cognitive changes, assessed through multimodal neuroimaging and electrophysiological measures. Resting-state functional MRI will be used to evaluate alterations in structural and functional connectivity within the default mode network (DMN), parietal memory network (PMN), and hippocampal regions, alongside entropy measures (Shannon and permutation entropy). Quantitative Electroencephalogram (EEG) will be used to capture changes in electrophysiological activity, including power spectral density, coherence, and network connectivity indices. These secondary measures will provide connection insights into the network-level effects of tDCS and their relationship to cognitive outcomes.

### Study design

**Figure 1:**
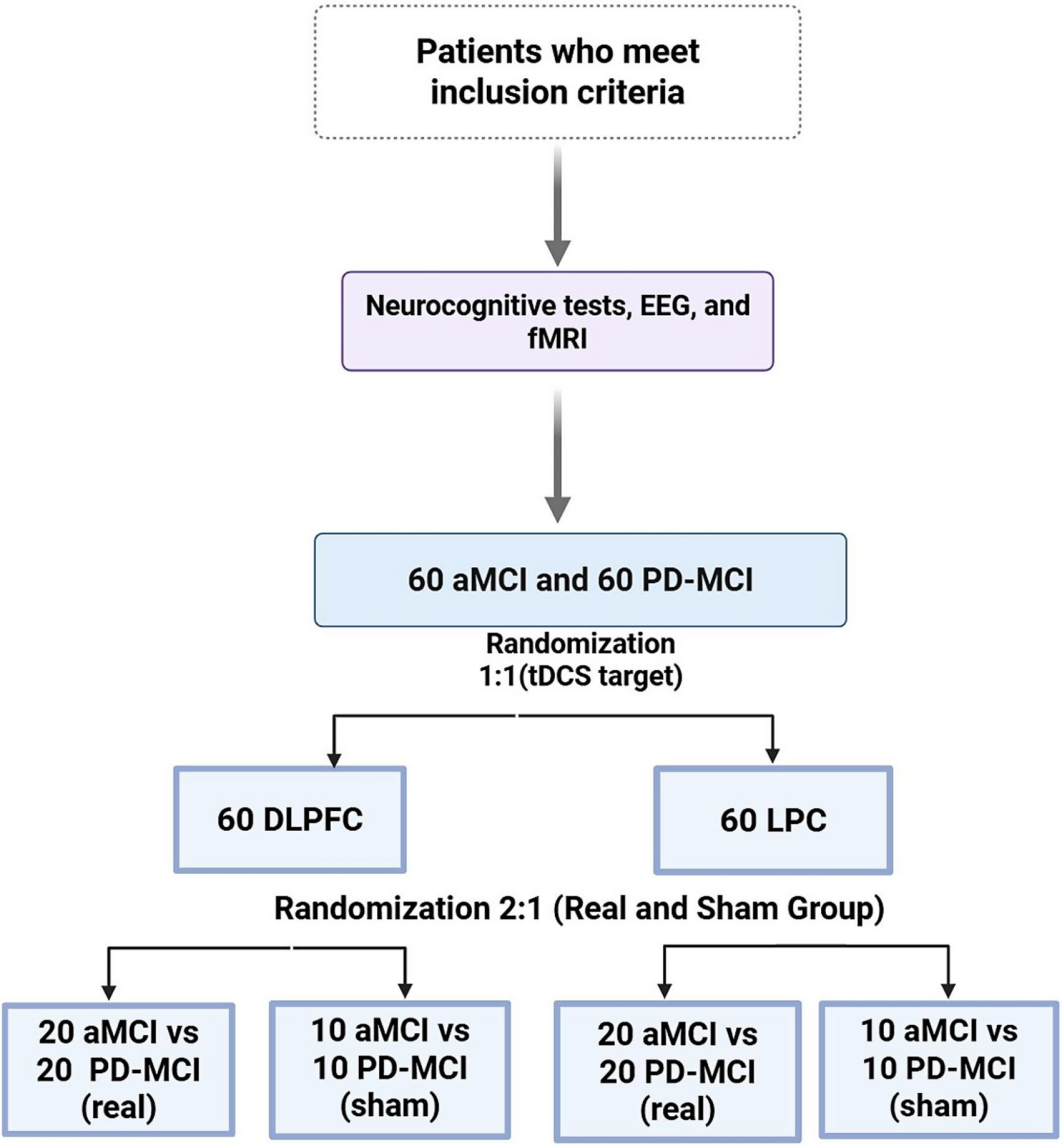
Study design and group allocation. Flowchart illustrating the study design for participants meeting inclusion criteria. Initial neurocognitive assessments, EEG, and fMRI were performed on 60 individuals with aMCI and 60 with PD-MCI. Participants were randomized in a 1:1 ratio to receive tDCS targeting either the DLPFC or the LPC. Within each stimulation arm, participants were further randomized in a 2:1 ratio to the Real or Sham tDCS condition, yielding 20 aMCI and 20 PD-MCI (real) versus 10 aMCI and 10 PD-MCI (sham). Created with BioRender.com.

This multi-center, randomized, double-blind controlled trial (ClinicalTrials.gov Identifier: NCT05919485) aims to evaluate the efficacy of anodal tDCS applied over the DLPC and LPC in improving cognitive performance in patients with PD-MCI and AD-MCI (Figure 1). All participants will be informed of experimental goals of this study. Written informed consent will be obtained. This study protocol was approved by Istanbul Medipol University Ethics Committee (No:161/16.02.2023) and complied with ethical standards based on the Declaration of Helsinki.

### Participants

The randomly generated group assignment list will be produced by statisticians not involved in data collection, and participants with either amnestic MCI (*n* = 60) or PD-MCI (*n* = 60) will be assigned in a 1:1 ratio to either the DLPFC or the LPC group. As a second step, the device administrator will configure the device according to the assignment list based on participants’ registration numbers, which will be set to either sham or active mode via the administration menu. Finally, 30 PD-MCI and 30 AD-MCI patients will receive either real or sham stimulation in a 2:1 ratio (Figure 1). Since only the device administrator, who will not interact with the participants, is aware of the assignment list and the password needed to access the administration menu, other investigators and participants will remain blinded.

**Figure 2:**
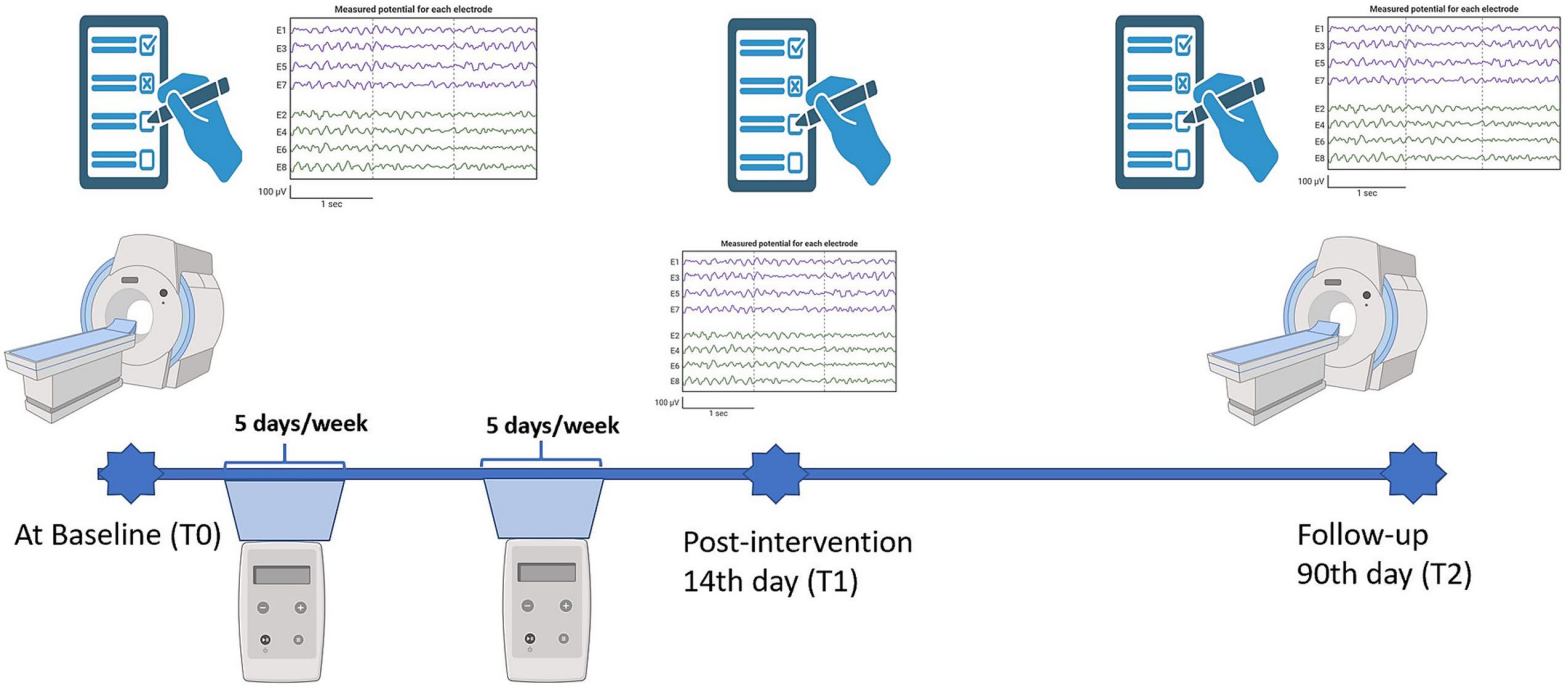
Timeline of the tDCS intervention protocol. Assessments were conducted at three time points: baseline (Day 0, T0), post-intervention (Day 14, T1), and follow-up (Day 90, T2). Cognitive testing and electroencephalography (EEG) were performed at each time point, while functional magnetic resonance imaging (fMRI) was performed at baseline and follow-up. Participants received tDCS sessions five days per week between baseline and post-intervention. Created with BioRender.com.

All participants will receive 2 mA tDCS in one session per day, administered over 10 days within 2 weeks, followed by a 90-day post-intervention assessment (Figure 2). For enrolment in the study, participants will be assessed using inclusion and exclusion criteria, accompanied by the Mini-Mental State Examination (MMSE), Montreal Cognitive Assessment (MoCA), and Clinical Dementia Rating (CDR) to evaluate dementia severity.

### Inclusion criteria

Selection criteria are: (1) Literate and between 45 and 80 years of age; and (2) Score of 19–23 points for MMSE and <26 points for MoCA; (3) clinical diagnosis will be conducted by a skilled neurologist using DSM-V (Diagnostic Statistical Manual of Mental Disorders, fifth edition) criteria for mild neurocognitive disorder.

### Exclusion criteria

Exclusion criteria are as follows: (1) history of dementia or intellectual disability; (2) use of cognitive enhancers (donepezil, rivastigmine, galantamine, and memantine); (3) reversible medical conditions associated with potential cognitive impairment (e.g., history of vitamin B12 deficiency or hypothyroidism); (4) history of neuropsychiatric disorder such as cerebrovascular surgery, brain trauma, demyelinating diseases, epilepsy, bipolar disorder, major depressive disorder, schizophrenia, anxiety disorders, and substance abuse; (5) Having medical issues that prevent undergoing fMRI or for tDCS application; (6) Illiterate or education level less than primary school.

### Procedures

#### Assessment procedures

Neuropsychological testing will be conducted at baseline, after the intervention (at 14th day), and at follow-up (at 90th day). The test batteries listed below have already been used in a comparison study about PD-MCI vs. AD-MCI.

- Attention: Digit Span Forward and Backward (Wechsler Memory Scale–Revised, WMS-R)
- Processing speed: Digit Symbol (Wechsler Adult Intelligence Scale–Revised, WAIS-R), Trail Making Test Part A
- Executive function: Trail Making Test Part B
- Episodic memory: Immediate and Delayed Logical Memory (WMS-R)
- Language: Animal fluency, Vegetable fluency, Boston Naming Test (BNT)
- Visuospatial ability: MMSE pentagon score (1 ¼ correct, 0 ¼ incorrect) and through clock-drawing (3 points), and cube copy (1 point) in MOCA.
- The Clinical Dementia Rating–Sum of Boxes (CDR-SB) will be completed at each visit. The CDR sum of boxes (CDR-SB) is a summary measure of scores for memory, orientation, judgment, community affairs, home and hobbies, and personal care, and ranges from 0 (no impairment) to 18 (greatest impairment).

#### Intervention procedures

The electrode will be located by placing the anode on the left DLPFC (F3), as positioned in the 10–20 international EEG system, and the cathodal electrode will be positioned over the contralateral supraorbital zone. In the LPC stimulation group, one electrode was positioned over P3 while the cathodal electrode was placed on the contralateral supraorbital zone.

The current density will be 0.06 mA/cm^2^ from each electrode with total density of 0.054/cm2 and will be delivered for 20 min a day for 10 days over 14 days.

For the sham protocol, stimulation will be delivered once with a very low current frequency, sufficient to cause slight tingling, and will last 15 s, with a total duration of 20 min. The number of sessions and days will be the same as the real group.

Electrode placement will be individualized using SimNIBS (Saturnino et al., 2019) software to optimize current flow targeting.

A safety strategy for tDCS will be applied throughout the whole procedure in the hospital by the same skilled investigator. Medical issues, including scalp malformation, inflammatory reaction, or other dermatological problems, will be examined by researchers during each visit.

#### Neuroimaging

Before the first tDCS stimulation session, participants underwent resting-state functional MRI (fMRI) data acquisition, which was repeated immediately after the completion of the stimulation series, following the 90^th^ day.

fMRI will evaluate structural and functional connectivity, with a specific focus on the default mode network (DMN), parietal memory network (PMN), and hippocampal connectivity. Network entropy will be computed using both Shannon and permutation entropy approaches.

R4 In detail, the processing step of the structural and functional connectivity will follow using the FreeSurfer (2025) and CONN Toolbox (2025). EEG pre-processing and connectivity analyses will follow standardized pipelines using open-source software (e.g., EEGLAB). Given the previous literature indicating the role of underlying connectivity and electrophysiological features of non-impaired structural brain regions in early dementia, we will pay special attention to the connectivity analysis. This will be assessed both at the ROI-to-ROI level and through whole-brain network analyses using the CONN Toolbox, with a focus on the parietal memory network (PMN) and default mode network (DMN). Entropy measures will be derived from resting-state correlation matrices to capture network complexity and integration. The calculation for different types of entropies will be performed according to the previously defined codes by Rostaghi and Azami (2016), which have already been used in our previous study (Yulug et al., 2025). The correction for multiple comparisons, family-wise error correction, will be applied, while for ROI-based analyses, correction will be used across the predefined set of regions to minimize false positive values.

### EEG (electroencephalogram)

*EEG Power Spectrum Analysis;* EEG data will be separated into one-second epochs after they are cleared of noise. Power spectrums of these data will be obtained in the delta, theta, alpha, beta and gamma frequency bands. Each epoch will be analyzed using the Fast Fourier Transform (FFT) with a 10% Hanning window. Subsequently, power spectrum analysis will be performed to obtain the frequency values for each electrode by averaging all FFTs. Maximum peaks will be determined in the delta (0.5–3.5 Hz), theta (4–7 Hz), alpha (8–13 Hz), beta (15–28 Hz), and gamma (28–48 Hz) frequency bands. These values will be used in statistical analysis for each person and electrode.

*EEG Coherence Analysis:* Coherence measurements at delta, theta, alpha, beta, and gamma frequencies can be analyzed for either intra-hemispheric electrode connections or inter-hemispheric electrode connections. Coherence values take values between 0 and 1. Values close to 0 indicate that there is no connection at the determined frequency between the two calculated electrode regions. In comparison, values close to 1 indicate a high coupling between the two electrode regions. Coherence calculation will be performed using the Brain Vision Analyzer program.^1^ The data obtained during memory and visualization tasks will be separated into one-second epochs after being cleared of noise. Power spectrums of these data will be obtained in the delta, theta, alpha, beta and gamma frequency bands. Fast Fourier Transform will analyze each epoch with a 10% Hanning window. Then, these data will be calculated for all possible electrode pairs using the Brain Vision analysis program with the formula given below.

*EEG functional connectivity analysis:* eLORETA software will be used for functional connectivity analysis. sLORETA/eLORETA is an online, ree software developed by Roberto Pascual-Marqui and his team^2^ (Pascual-Marqui et al., 1994). eLORETA is an algorithm developed to solve the inverse problem and it does not contain localization bias even in the presence of noise. In this software, resting state data with eyes closed, separated into 2-s epochs, whose artifacts are cleaned by pre-processing, will be used. The relevant areas to be analyzed in the cortical plane and the relevant frequency band gaps will be determined. The time series containing the eLORETA current source density obtained from these areas will be calculated, and a “lagged linear coherence” matrix will be created to be applied in graph theory (Vecchio et al., 2016). “Lagged linear coherence” will give correct physiological connectivity, unaffected by volume conduction and low spatial resolution.

### Statistical analyses

All statistical analyses will be conducted using IBM SPSS Statistics, version 29.0 (IBM Corp., Armonk, NY, United States).

Analyses will be performed stratified by diagnosis (AD-MCI and PD-MCI):

*Neurocognitive outcomes:* Within each diagnosis group, repeated-measures ANOVA/GLM will be used with Time (Baseline, Day 14, Day 90) as a within-subject factor, Group (Real vs. Sham) and Target (DLPFC vs. LPC) as between-subject factors, and age, sex, education, and baseline global cognition as covariates. Planned contrasts will test the Group × Target × Time interaction separately in AD-MCI and PD-MCI. Between-diagnosis comparisons will then examine whether the effect of stimulation differs between AD-MCI and PD-MCI by including Diagnosis (AD-MCI vs. PD-MCI) as an additional between-subject factor. Post-hoc tests will be adjusted with Bonferroni correction.

*fMRI outcomes:* Resting-state fMRI data will be analyzed separately within AD-MCI and PD-MCI groups to assess functional connectivity within DMN, PMN, and hippocampal regions, as well as entropy metrics (Shannon and permutation entropy). Longitudinal changes (Baseline, Day 14, Day 90) and group differences (Real vs. Sham; DLPFC vs. LPC) will be assessed using repeated-measures ANOVA/ANCOVA with the same covariates.

After stratified analyses, diagnosis will be included as a factor to test for Diagnosis × Group × Target × Time interactions.

### Power calculation

The sample size was informed by previous studies applying tDCS in populations with cognitive impairment. For example, Khedr et al. administered active and sham tDCS to the left DLPFC over 10 consecutive days in 34 participants (Khedr et al., 2014). Additionally, the same group has shown efficacy of tDCS with the anode (positive electrode) placed at the T3-P3 or T4-P4 in 46 AD patients (Khedr et al., 2019).

Based on these precedents, and assuming a medium effect size (Cohen’s d ≈ 0.5–0.6) as reported in prior MCI/AD and motor adaptation studies, we calculated that a minimum of 50 participants per group would be required to achieve 90% power (1–*β* = 0.9) at a two-tailed *α* = 0.05. To allow for an anticipated dropout rate of approximately 20%, we increased the target to 60 participants in each diagnostic group (AD-MCI and PD-MCI), yielding a total sample of 120 participants. This sample size ensures sufficient power to detect clinically meaningful differences in cognitive outcomes between stimulation conditions.

## Discussion

Our hypothesis, which aims to determine whether region-specific tDCS may exert significant pro-cognitive effects when applied during the early stages of Parkinson’s and Alzheimer’s dementia, is deserving of mention given the existing literature on the effects of tDCS. Herein, there is valuable data that discusses the influence of tDCS on cognition in both healthy and pathological conditions, emphasizing principally the importance of stimulation location and disease stage (i.e., PD-MCI or AD-MCI).

Numerous studies imply that MCI is a common intermediate stage for the development of various types of dementia. One advantage of this shared stage is the potential to offer a two-for-one strategy that could benefit from novel neuromodulating methods such as tDCS, which may be essential for filling an inevitable gap in the early management of progressive dementia diseases. tDCS application over DLPFC has been already shown to augment functional connectivity between DMN and frontoparietal regions while simultaneously causing decreased DMN activity during the performance of a cognitive task associated with relevant resting EEG changes (Marceglia et al., 2016; Vulić et al., 2021) that resulted in improved motor and cognitive functions (Brak et al., 2022; Ferrucci et al., 2008). Furthermore, stimulation of temporoparietal regions resulted in enhanced performance in memory and global cognitive functions (Park et al., 2019), leading to a comparable level of improvements obtained from pharmacological treatment using cholinesterase inhibitors (Ferrucci et al., 2008; Khedr et al., 2019; Marceglia et al., 2016). These findings were suggested with fMRI studies with anodal tDCS showing considerably increased connectivity between DMN nodes (hippocampus, dorsal attention network, CN, salient network, and sensory-motor network) (Manenti et al., 2010, 2020), and increased segregation in DMN networks (Hunter et al., 2015).

In addition to the role of DMN, PMN seems to be a promising target for the diagnosis and treatment of cognitive impairment. Although PMN is anatomically and functionally adjacent to the DMN, evidence suggests its distinct role as a functional network consisting of parietal regions, cingulate, and the precuneus (Gilmore et al., 2015). Furthermore, in contrast to the DMN, which is more commonly implicated in self-referential, and mind wandering (Sestieri et al., 2011) accumulating evidence suggest that PMN is involved in specific cognitive functions such as episodic and associative memory processing (Hu et al., 2016). Hence, targeting the PMN might be a unique stimulation approach, especially when considering recent evidence that the PMN integrity is impaired in early stages of dementia development (Velioglu et al., 2021; Vulić et al., 2021).

In accordance with these findings, the restoration of impaired functional integrity between LPC and hippocampal regions resulted in an improved performance on recognition memory after repetitive magnetic stimulation (rTMS), in Alzheimer’s patients (Velioglu et al., 2021). These findings were aligned with tDCS stimulation of the posterior parietal cortex (PPC), resulting in improved performance that persisted even 5 days after stimulation (Vulić et al., 2021).

These results affirmed by the results of a recent meta-analysis covering more than 200 healthy subjects, suggesting that PPC (Galli et al., 2019) and PMN-targeted stimulation exerted considerably higher pro-cognitive effects than other application sites, which may be related to unique connectivity changes between the precuneus, lingual gyrus, and hippocampus in healthy subjects (Warren et al., 2019). Also, the following studies evaluating the effect of parietal stimulation through tDCS gave promising results. For instance, Zivanovic et al. have revealed that different types of tDCS applied on the parietal region revealed comparable beneficial effects on short term associative memory (Živanović et al., 2022). The role of tDCS on specific memory domains has been also suggested by a very recent study by healthy individuals showing that the stimulation of parietal cortices resulted in improved verbal memory associated with theta and alpha power changes on responsible brain regions (Arif et al., 2024).

This study has some limitations. Firstly, despite large number of participants, given the multiple groups, it is a small-scale prospective study necessitating a study with a larger number per group to substantiate the clinical efficacy. Secondly, the small number of sessions and indirect stimulation of hippocampal circuits, along with the heterogeneity caused by clinical presentation due to different diseases (PD-MCI and AD-MCI), limit the generalizability of our findings. It is also worth discussing our study design, which proposes shorter courses of tDCS application compared to the previous literature. Herein, our design was principally based on the literature demonstrating that shorter interventions can also be effective, allowing us to optimize the balance between scientific rigor and patient adherence. In that context, while some studies have used longer protocols (20–30 sessions), there is also solid evidence that shorter courses ranging between one and 10 sessions (Khedr et al., 2019; Rasmussen et al., 2021; Smirni et al., 2021) can already produce meaningful cognitive and neural improvements (Prathum et al., 2025). For instance, Smirni et al. have shown recovery on phonemic fluency performance even after one session (Smirni et al., 2021) while Kehdr et al. have shown the improvement of cognitive test scores after 10 sessions of tDCS over 2 weeks (Khedr et al., 2019). The impact of shorter courses of tDCS on cognitive functions was suggested with the following study by Rasmussen et al. showing that 6 sessions (3 sessions/day, 2 days) was effective on delayed memory impairment in patients with AD (Rasmussen et al., 2021).

We therefore selected 10 days over 14 days as a practical and participant-friendly schedule, which is easier for patients to complete, helps minimize dropout, and makes the intervention more feasible in real-world clinical settings.

Nevertheless, caution should be exercised when applying this procedure to patients with early cognitive impairment especially regarding the efficacy of tDCS, which may depend on the indirect nature of the stimulus that might not reach the hippocampus. Within that context, despite some positive results, including also our previous study (Velioglu et al., 2021), the inducibility of the hippocampus through stimulation of the parietal cortex target areas will be tested concerning dosages with varying intensities to achieve the optimal effect of tDCS on cognitive symptoms, alongside changes in hippocampal connectivity.

To sum up, considering its beneficial cognitive effects in healthy individuals, tDCS is not only advantageous for early intervention but also for understanding the ethology and pathogenesis of early stages of dementia such as in AD-MCI and PD-MCI, as it has the potential to provide a clearer link between neurostructural and functional pathologies and neurocognitive outcomes. The application of tDCS to unconventional regions, combined with detailed fMRI analyses, will yield new insights into the pathophysiology of these diseases.

## Conclusion

The restorative benefits of tDCS can be critical to understanding the cause-effect relationship between certain neural circuits and behavioral outcomes, which may be significant in illuminating the early pathophysiology of AD and PD. From another perspective, our findings may also be valuable in terms of prolonged life expectancy and the maintenance of preserved cognitive skills in the elderly. Although tDCS is still in a very early stage, it offers several advantages in terms of portability, cost-effectiveness, and user-friendly application over other non-invasive stimulation applications.

## Footnotes

^1^BrainVision Analyzer (Version 2.), Gilching, Germany: Brain Products GmbH.

^2^ http://www.uzh.ch/keyinst/loreta.htm
